# Cybersecurity and Cyber Forensics for Smart Cities: A Comprehensive Literature Review and Survey

**DOI:** 10.3390/s23073681

**Published:** 2023-04-02

**Authors:** Kyounggon Kim, Istabraq Mohammed Alshenaifi, Sundaresan Ramachandran, Jisu Kim, Tanveer Zia, Abdulrazaq Almorjan

**Affiliations:** 1 Center of Excellence in Cybercrime and Digital Forensics, Naif Arab University for Security Sciences, Riyadh 14812, Saudi Arabia; 2 BoB (Best of the Best), Korea Information Technology Research Institute, Seoul 08378, Republic of Korea

**Keywords:** smart city, cybersecurity, digital forensics, cyber forensics, Internet of Things

## Abstract

Smart technologies, such as the Internet of Things (IoT), cloud computing, and artificial intelligence (AI), are being adopted in cities and transforming them into smart cities. In smart cities, various network technologies, such as the Internet and IoT, are combined to exchange real-time information, making the everyday lives of their residents more convenient. However, there is a lack of systematic research on cybersecurity and cyber forensics in smart cities. This paper presents a comprehensive review and survey of cybersecurity and cyber forensics for smart cities. We analysed 154 papers that were published from 2015 to 2022 and proposed a new framework based on a decade of related research papers. We identified four major areas and eleven sub-areas for smart cities. We found that smart homes and the IoT were the most active research areas within the cybersecurity field. Additionally, we found that research on cyber forensics for smart cities was relatively limited compared to that on cybersecurity. Since 2020, there have been many studies on the IoT (which is a technological component of smart cities) that have utilized machine learning and deep learning. Due to the transmission of large-scale data through IoT devices in smart cities, ML and DL are expected to continue playing critical roles in smart city research.

## 1. Introduction

Many countries are exploring the implementation of smart cities to provide better lives for their citizens. The creation of smart and sustainable cities is enabled by current and emerging technologies, such as the Internet of Things (IoT), sensors, artificial intelligence (AI), robotics, unmanned systems, digital twins, Web 3.0 technologies, and smart grids. The rapid penetration of smartphones and information and communication technology (ICT) has been a fundamental enabler of smart cities, providing ubiquitous connectivity for smart city technologies and helping to improve connectivity, share information with the public, and provide better quality government services.

In addition, many countries are making efforts to transform certain cities into smart cities, including Dubai, Tokyo, Singapore, Hong Kong, Seoul, Helsinki, London, and Riyadh [1,2]. In particular, Saudi Arabia is investing 5 billion USD in the construction of NEOM, a new smart city [3]. The city of Riyadh in Saudi Arabia uses anti-congestion measures for traffic, including adaptive traffic control. The city’s traffic is monitored and managed by an intelligent transport system (ITS) that uses a variety of sensors and an advanced CCTV surveillance system. To inform traffic dashboards, advanced analytics are used to undertake historical, real-time, and predictive traffic analysis, including contextualized incident and traffic reporting [4].

Singapore is also striving to become a smart city in order to enhance the living environments of its residents [5]. Virtual Singapore is a dynamic 3D city model and collaborative platform created by Singapore to assist city stakeholders in promoting this innovation. Using the Virtual Singapore program, city stakeholders can gain valuable insights for policy and business research, decision-making, and prototyping [4].

The intelligent city platform of Valencia (VLCi) is a cloud-based internal city management system created in Spain. It enables the city to gather crucial information on its urban services, analyse that information using cutting-edge tools, and create dashboards to assist with decision-making [4]. The city of Texel in the Netherlands has recently upgraded to sophisticated, energy-saving street lighting equipment. By combining LEDs and IoT sensors, the city has significantly reduced energy consumption and light pollution [4]. London is also exploring changes to its smart city approach, based on active participation and feedback from its citizens [6].

Cities are constantly striving to make the lives of their citizens more convenient. Not only are traditional cities being transformed into smart cities but new cities are being constructed as smart cities. Smart cities have the potential to improve urban life through efficient transportation, energy efficiency, and improved public services. However, concerns around data privacy, social equity, dependence on technology, and environmental impacts must be addressed to ensure that smart cities can truly benefit all citizens and the environment.

Many new technologies are being applied to facilitate the transition to smart cities, but these can also pose potential risks. Smart homes offer convenient features through the use of new technologies, such as the IoT, but there are also vulnerabilities that hackers can exploit to remotely control smart homes. One experiment highlighted the vulnerability of the Z-Wave technology that is used in smart homes, in which a smart door was opened remotely and the smart valve alert was disabled, leaving the user unaware of a fire in the house [7,8]. Smart mobility provides freedom of movement for residents in smart cities, but it also poses a threat to life if it falls into the hands of attackers [9]. At the Black Hat conference, researchers successfully demonstrated that an autonomous vehicle could be hacked remotely and the brakes forcibly activated while driving [10]. Therefore, if a core element of a smart city is targeted by a cyberattack, it could result in significant damage to life and property.

State-sponsored hackers who employ advanced technologies, such as ransomware, have emerged as significant threats to smart cities [11,12]. Attacks on the core elements of smart cities not only benefit cyberattackers but also cybercriminals. By exploiting the vulnerabilities in smart homes, cybercriminals can remotely open doors or operate smart car keys to gain unauthorized access. In such cases, it is difficult to determine whether an attack was performed by a cybercriminal or a legitimate user. Therefore, further research on the use of cyber forensics to investigate cybercrimes in smart cities is critical.

Given the significance of smart cities, many researchers have been investigating their security and forensics. Research on smart cities has focused on various aspects. For example, Losavio conducted a study on the legal challenges of smart cities and IoT technologies, focusing on digital forensics, privacy, and security perspectives [13]. Baig et al. conducted a study on the future challenges of key elements in smart cities, including smart vehicles, unmanned aerial vehicles (UAVs), building automation systems (BASs), and smart grids [14]. Despite the significance of smart cities, there has been a lack of comprehensive research on identifying their key elements and addressing the challenges in cybersecurity and cyber forensics.

This study focused on reviewing the literature from 2015 to 2022 and analysing cybersecurity and cyber forensics research related to smart cities. To identify the key elements of smart cities, we conducted an initial search for related papers using relevant keywords. Then, for each key element, we systematically reviewed the related literature on cybersecurity and cyber forensics. The following keywords were used to search in Google Scholar: “cybersecurity smart cities”, “cyber forensics smart cities”, “digital forensics smart cities”, “smart home”, “smart mobility”, “smart vehicle”, “smart things”, and “smart people”. Additional recently published papers focusing on cybersecurity and cyber forensics were also selected.

This review is intended for readers who are conducting research in the areas of cybersecurity and cyber forensics for smart cities. The contributions of this paper include the following:

• A comprehensive review of 154 papers on cybersecurity and cyber forensics for smart cities that were published from 2015 to 2022, which revealed the key areas of research and identified major challenges and opportunities for improving the cybersecurity and cyber forensics readiness of smart cities;
• A chronological organization of the research on cybersecurity and cyber forensics for smart cities, based on the key technologies that have been studied over time, such as the IoT, cloud computing, and AI, in order to provide a concise overview of the evolution of research on cybersecurity and cyber forensics for smart cities;
• A comprehensive investigation of cybersecurity in smart cities, which found that the most common targets of cyberattacks are smart homes and that attacks related to the IoT are being studied very actively;
• The observations that compared studies on cybersecurity in smart cities. There have been relatively few studies related to cyber forensics and that among the key city elements of smart cities, research on cyber forensics for IoT devices has been the most common.

The remainder of this paper is organized as follows. Section 2 discusses the key components of smart cities, including smart homes, smart vehicles, smart factories, and smart people. In Section 3, we provide a summary of cybersecurity research papers that were relevant to smart cities. Section 4 describes the research on cyber forensics that pertained to smart cities. Section 5 addresses the future expectations and research directions to smart cities. Finally, in Section 6, we present our observations and conclusions.

## 2. Key Elements of Smart Cities

**Figure 1:**
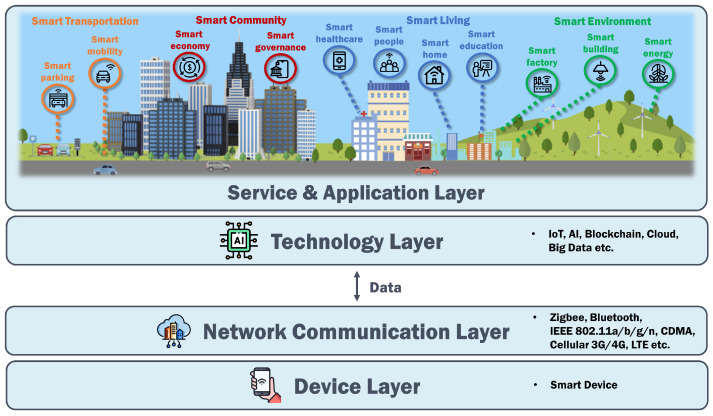
The architecture of smart cities. All elements were identified by the authors. The layers and area icons of smart cities were made using Freepik, orvipixel, Mayor Icons, Barudaklier, and KP Arts from www.flaticon.com, accessed on 29 March 2023 [22].

To investigate and study smart cities, it was essential to identify their key elements. For this purpose, we used keywords, such as “smart city elements”, “smart city layers”, “smart city architecture”, and “smart city ecosystem”, to identify the key elements of smart cities. As shown in Table 1, several authors have attempted to identify the key elements of smart cities. By analysing the papers found using these keywords [15,16,17,18,19,20,21], we derived the core elements of smart cities, as shown in Figure 1.

As depicted in Figure 1, smart cities can be divided into four layers. In the top layer, there is the provision of services and applications for residents living in the smart cities. Below this, there are the technology, network communication, and device layers.

Within the service and application layer, four key sub-areas were identified: smart transportation, smart communities, smart living, and smart environments. The smart transportation area comprises smart parking and smart mobility, while smart communities include smart economy and smart governance. Smart living can also be further divided into sub-areas, which include smart healthcare, smart people, smart homes, and smart education. Lastly, smart environments can be classified into smart factories, smart buildings, and smart energy.

### 2.1. Service and Application Layer

Smart city services and applications aim to enhance the quality of life of residents in smart cities. These services enable citizens to use urban services efficiently and conveniently by utilizing advanced technologies.

Smart transportation systems offer a range of services, from guiding users to better routes or sharing traffic conditions to car and bike sharing opportunities, making public transportation systems and citizen mobility more efficient and convenient. Additionally, smart services can also cater to the parking needs of citizens, such as the identification of available parking spaces [23].

From the governmental perspective, ICT can be used to promote transparency and community participation and effectively manage crowds and solve emergency problems based on public data [20]. Smart economy enhances the flexibility of the labour market, as well as regional productivity and competitiveness.

Smart living services allow citizens in smart cities to share information in real time, thereby enabling them to efficiently solve everyday challenges and stay connected [20]. Remote health monitoring and e-health services provide citizens with customized health and medical care [24].

Smart home systems allow users to remotely control IoT home appliances, such as smart TVs, by using smartphone applications to perform specific tasks [25]. Additionally, urban facilities can be utilized more conveniently and education systems can be improved to provide flexible education options for residents.

One of the key goals of smart cities is to promote environmental sustainability through digitally integrated and improved energy management systems. Smart services can carry out the real-time monitoring and management of smart buildings, smart factories, and smart energy systems, which can enhance energy efficiency and waste management [20].

Cyberattacks are primarily directed towards smart city services and applications, making cybersecurity countermeasures essential for ensuring the safety of smart cities. The risk of cybercrime also increases with the implementation of these technologies. To address cybercrime, policing must also evolve in the future, with data analysis playing a central role in this evolution [26]. Research is also being conducted on data-driven policing for smart cities. In addition, current and developing technologies have been analysed, along with the opportunities they provide for smart policing in smart cities [26].

Section 2.1 discussed the service and application layer of smart cities, which aims to improve the quality of life of citizens by providing efficient and convenient urban services. Through our investigation of the key elements of smart cities, we identified four major categories and eleven sub-categories in the service and application layer, such as smart transportation, smart communities, smart living, and smart environments.

### 2.2. Technology Layer

Innovative and popular technologies play crucial roles in more efficiently and safely solving various problems with smart cities by utilizing the vast amounts of data that are generated. The IoT is a fundamental technology that connects various ICT devices and lays the foundations for advanced smart cities [27]. Research has been conducted on applying IoT technologies to various smart city applications, including pollution issues [28,29,30]. Cloud infrastructure and platforms are also crucial in smart cities and data separation is a significant concern [31,32]. Data can be collected in real time using sensors in IoT devices, such as RFID, infrared radiation (IR), global positioning systems (GPSs), and laser scanners, and stored in the cloud or edge data storage. IoT applications enable the remote control and management of devices [27,33].

AI technology is rapidly evolving to enable the fast and efficient delivery of complex services for small-scale smart city elements, such as smart homes and smart buildings, as well as large-scale elements, such as smart infrastructure and smart transportation [34]. Big data technology is a key enabler of AI as it can collect and analyse the vast amount of data generated in smart cities, thereby allowing AI to predict or infer future results and make better decisions. Additionally, blockchain and deep learning technologies can be used to provide smarter automation services for smart cities as data can be learned and determined autonomously [35]. Authentication technology is also being researched for smart cities from a blockchain perspective [36].

However, privacy and security issues arise when data are exchanged across a wide range of areas in smart cities. As a potential solution, blockchain is increasingly being used for some data exchanges. By enabling smart transactions through smart contracts and decentralized applications, blockchain provides a high degree of autonomy for the operation of smart cities [37]. Additionally, research is being conducted on cybersecurity issues related to the use of augmented reality (AR) technologies in smart cities [38].

User perceptions of smart city techniques depend on various factors, such as personal experiences, data privacy, and data security. Users are more likely to perceive smart city techniques positively when they see clear benefits, such as enhanced public services. A study conducted by Silver Spring Networks found that 75% of US consumers had a positive view of smart cities after being educated about their benefits, while only 3% had a negative view. The top two benefits perceived by respondents were reducing pollution and enhancing public safety, while the top two concerns were costs and privacy. Additionally, the study found that positive sentiments towards smart cities were expressed as an interest in living with smart city technologies.

Section 2.2 highlighted the use of the IoT and cloud infrastructure for data collection, storage, and management, as well as the roles of AI and big data technology in providing advanced services via prediction and inference. Blockchain technology was also introduced as a potential solution for privacy and security concerns in smart cities as it provides a high degree of autonomy when executing smart transactions. However, due to the increasing use of technologies in smart cities, cybersecurity countermeasures are necessary to prevent cyberattacks and cybercrime.

### 2.3. Network Communication Layer

Network communication is a crucial element in smart cities. Applications that require communication over short distances, such as smart grids, smart water services, and smart buildings with very limited energy usage, typically utilize the IEEE 802.15.4 (Zigbee). These applications can last for years using the same battery. IEEE 802.15.1 (Bluetooth) can also be used for these applications. The wireless local area network (WLAN) protocol uses the 2.4 GHz band, with a data rate of 1 Mbps and a master/slave time division duplex (TDD) ranging from 10 to 100 m.

The IEEE 802.11a/b/g/n protocols are used in almost all smart city systems. The latest version, the IEEE 802.11n protocol, operates in the 2.4 and 5.1 GHz ranges and uses orthogonal frequency division multiplexing (OFDM) and a direct sequence spread spectrum (DSSS). This protocol also enables reservation-based operations using point coordination functions (PCFs) and best effort operations using distributed coordination functions (DCFs). PCF services are useful for video, real-time, and multimedia audio data traffic that requires QoS guarantees for specific parameters, such as bandwidth, delay, and delay jitter [39].

Smart water services, UAVs, smart grids, and pipeline monitoring can use cellular 3G and 4G protocols that employ packet switching for data communication and circuit switching or selective packets for voice communication [39]. Long-term evolution (LTE), which is an advanced technology, can also be used and coverage is available worldwide when roaming is employed.

UAVs, pipeline monitoring, and smart transportation can also utilize satellite communication, which typically uses frequencies in the 1.53 and 31 GHz ranges and employs time division multiple access (TDMA) and frequency division multiple access (FDMA) at the data link layer [39]. It also has data rates of 1 Gbps (upload) and 10 Mbps (download), making continuous coverage possible due to the trade-off between satellites worldwide [39].

The efficient spectra of narrow-band technologies are suitable for smart cities [40] as they can support several IoT devices in smart grids, smart homes, and smart meters, among others [40]. However, despite their advantages, narrow-band technologies have limitations regarding security, which can make them vulnerable to cyberattacks [40]. The information-centric networking (ICN) routers can be used in smart home networks for more efficient content distribution due to their increased caching capacity and improved cybersecurity [41].

It is essential to protect smart city wireless sensor networks (WSNs) and a comparative study on anomaly detection technology was conducted for this purpose [42]. Research on attacks on smart city WSNs is also active and frameworks for attack classification and defence have been developed [43,44].

Section 2.3 discussed the various communication technologies that can be used in smart cities. These include IEEE 802.15.4 (Zigbee) and IEEE 802.15.1 (Bluetooth) for short-distance applications, such as smart grids and smart buildings, IEEE 802.11a/b/g/n protocols for large-scale smart city systems, and cellular 3G and 4G protocols and satellite communication for UAVs, pipeline monitoring, and smart transportation.

### 2.4. Device and Sensor Layer

The device and sensor layer is an essential component of smart cities. Smart devices equipped with sensors and actuators can measure, collect, and control various types of data [45]. Data from across smart cities, including information on citizen movement, parking, and building or city data (such as light, noise, temperature, and humidity), can be collected using IoT sensors and processed by converging them with ICT [45].

Citizens can visually receive and control data through physical devices, such as wearable devices and smartphones. Facilities equipped with IoT sensors can monitor data in real time and analyse and control these data according to their own specific purposes.

Smart city sensing is a new paradigm that promotes the transition to smart city services. A number of studies have been conducted on current and historic smart city sensing and its influence on related issues [46].

While the mass proliferation of IoT devices has provided advanced services based on hyperconnectivity, end-node devices focus on confidentiality and have weak security [16], which can affect connected smart devices.

## 3. Cybersecurity in Smart Cities

Cybersecurity for smart cities includes a combination of technologies that have emerged to address the highly complex challenges of insecure devices and networks, which can lead to unbounded attacks. In this section, we highlight the issues discovered by research and their proposed solutions.

**Figure 2:**
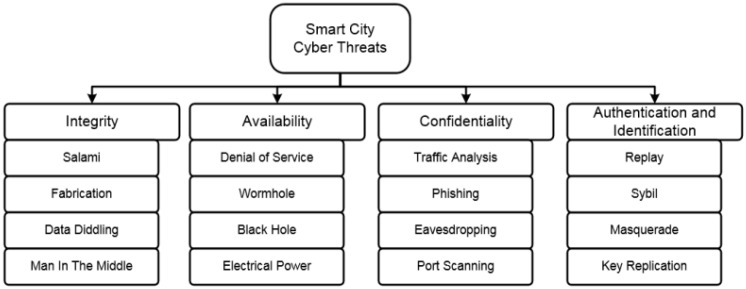
A common classification for the cyber threats of smart city infrastructure [47].

Several studies have been conducted on the cybersecurity challenges in smart cities. For example, Kalinin et al. [47] classified common cyber threats to smart city infrastructure, as depicted in Figure 2. AlDairi et al. [48] conducted a literature review of security and privacy issues in smart cities and proposed solutions to address them. Neshenko [49] designed a method to detect smart city industrial control system (ICS) attacks by investigating unnoticed changes in network traffic patterns and using interactive visualization to filter out false alarms. Alassad et al. [50] proposed a model to prevent damage from cyberattacks originating from online social networks using focal structure analysis (FSA) and deviant cyber flash mob detection (DCFM). Hamid and Bawany [51] presented a security framework called ACIDS, which identifies potential security threats in the five layers of smart city systems and helps to develop security measures in different fields.

Alibasic et al. [52] explained the main concepts of smart cities and their components. They also reviewed different cybersecurity threats and challenges and proposed solutions. Al-Turjman et al. [53] discussed the security and privacy issues of smart city applications, as well as their data-centric architecture, and addressed the main security issues of smart city architecture. Alamer and Almaiah [54] presented the challenges and threats of cybersecurity in smart cities and devised possible solutions. They also explained the advantages and opportunities of smart cities. Duan et al. [55] discussed large-scale video issues, as well as the future of using deep learning features to manage these issues in smart cities. Lakhouil et al. [56] explained the concepts, services, limitations, and investigations of ICT cybersecurity threats in smart cities. Furthermore, they discussed solutions to reduce the risks of these threats.

Ma [57] provided functional solutions for maintaining both user privacy and security in smart cities and also explained relevant cybersecurity threats. Figueiredo et al. [58] discussed the cybersecurity issues and risks in smart city operations. They also presented the current cybersecurity techniques and their applications and discussed possible future directions for smart cities. Wang et al. [59] proposed strategies for reducing cybersecurity attacks on smart city systems and protecting misused data. Furthermore, they demonstrated the highly effective mitigation of threats by following this approach. Biswas and Muthukkumarasamy [45] used smart devices with blockchain technology to secure data communication in a smart city. The main purpose of using blockchain is to improve resilience against cybersecurity threats and create secure communication among devices in disturbed environments.

Butt and Afzaal [60] discussed the security and privacy issues in smart cities and also investigated solutions that have been proposed in previous research. Furthermore, they identified the details of smart city applications and their vulnerabilities. Andrade et al. [61] analysed various issues and challenges with implementing IoT systems in smart cities and discussed the related security risks. Juma and Shaalan [62] reviewed cyber–physical system (CPS) trends in smart cities. They surveyed related works, discussed the challenges, and identified expected solutions from big data, the IoT, and cloud computing. They emphasized that these solutions could help to create significant impacts on smart cities and their applications. These studies highlighted the details of smart cities and provided valuable insights into improving the security of smart city systems.

In this section, we summarized the cybersecurity-related research on each service element within smart cities, as shown in Table 2.

### 3.1. Smart Transportation/Mobility

**Figure 3:**
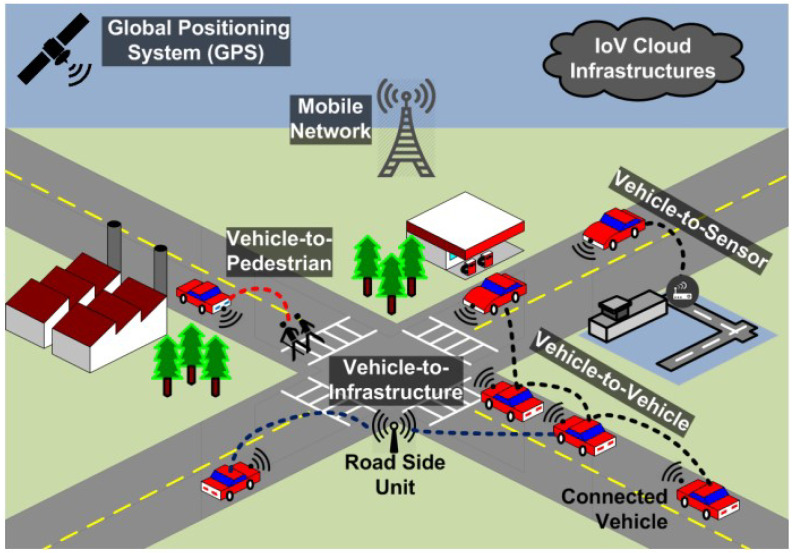
The ITS communication architecture in smart cities [63].

Javed et al. [63] conducted a security study on next-generation intelligent transport system (ITS) applications in smart cities, as shown in Figure 3. After analysing the security architecture of the European Telecommunications Standards Institute (ETSI) ITS standard, they implemented ECC-based digital signatures and encryption procedures using experimental test beds. In their study, a network simulation model was used to reproduce the smart city scenario. From the experimental results, they found that existing security solutions could directly affect the quality of service and safety perception of vehicle applications. Wang et al. [64] provided strategies to protect connected vehicles and the AI in the vehicles and also discussed automobile cybersecurity attacks. Kim et al. [9] discussed the implications of attacks on autonomous vehicles and how to defend against such attacks when integrated with the AI within the main components of smart cities, based on a systemic survey.

Sharmila et al. [65] analysed the different vulnerabilities and threats in autonomous vehicles and provided a model to mitigate security threats. Chen and Quan [66] discussed various attacks and targets within the Internet of Vehicles (IoV). They also proposed a framework for the IoV based on blockchain and suggested solutions for security, authentication, and privacy issues in the IoV.

ITSs and autonomous vehicles are essential components of smart cities. Researchers have analysed the security of these systems, including their architecture, vulnerabilities, and potential attacks. They have proposed various strategies to safeguard connected vehicles and mitigate security threats, including digital signatures and encryption procedures, as well as the use of blockchain frameworks. Studies have also shown that existing security solutions can affect the quality of service and safety perception of vehicle applications.

### 3.2. Smart Homes

Several research papers have addressed the cybersecurity and privacy concerns regarding smart homes. Ryu and Kwak [67] investigated the risk of unauthorized access to smart homes and proposed a secure data access control scheme to prevent data leakage, privacy invasion, and the falsification of data. McGee [68] evaluated the personally identifiable information (PII) vulnerabilities in smart home ecosystems and developed a security as a service (SECaaS) capability to assess the results. Liu and Hu [69] highlighted the cybersecurity vulnerabilities in smart home infrastructure, particularly energy bill scheduling techniques, and described current detection methods and cyberattack tools. Nsunza et al. [70] conducted an experiment to assess the performance of TCP and UDP network traffic using field programmable gate arrays (FPGAs) and system-on-chip (SoC) platforms in smart home routers.

Gamundani et al. [71] and Ghirardello et al. [72] investigated the vulnerabilities of authentication and home automation systems in smart homes and potential attack opportunities. Kraemer and Flechais [73] explored the future directions of privacy research within the smart home domain. Bastos et al. [74] proposed solutions for security issues in IoT devices in smart homes and predicted possible future cyberattacks. Sturgess et al. [75] identified three factors that contributed to smart home privacy risks. The first factor involved evaluating heterogeneous devices from a top-down perspective. The second factor concerned the various cyberthreats that exist in smart homes. The final factor explained the difficulties in aggregating the highly valuable private data of homeowners. They suggested a capability-oriented model to facilitate the rapid development of smart homes. Siddhanti et al. [76] suggested using a cybersecurity maturity assessment tool to secure smart home environments from cyberthreats.

Elmisery and Sertovic [77] suggested a permission-based approach for revealing log records that require involvement with third parties. Personal usage logs from homes that are shared with third parties can lead to attacks on smart home environments. Rossi et al. [78] identified a solution for detecting the exploitation of smart home systems by monitoring vulnerabilities in the systems-of-systems domain using a combination of defensive programming and Shodan APIs. Shodan APIs are a set of application programming interfaces (APIs) provided by the Shodan search engine that enable users to search and access information about Internet-connected devices and systems. Giannoutakis et al. [79] presented a framework to address this issue by using blockchain technology to ensure the integrity of smart home devices and block malicious IPs from accessing smart home environments.

In 2021, Rauti et al. [80] demonstrated attacks on the Chrome web browser by implementing a malicious browser extension. The user activities were modified, which affected the management consoles of smart homes. They showed that the connection of IoT devices to smart home networks caused potential man-in-the-browser attacks to target the remote control systems. Awang et al. [81] proposed solutions to enhance the IoT ecosystems in smart homes and analysed the possible threats to smart home operational environments. Turner et al. [82] discussed how the Internet connection between devices introduces security risks to smart homes as it allows access to private user information. They also provided recommendations for users regarding safely accessing cybersecurity data. Alshboul et al. [83] also proposed a methodology for detecting and predicting intruders attempting to recognize the identities of smart home sensors. They emphasized that data should preserve their identity by knowing their sources and not adding extra loads to the network.

In 2022, Mahor et al. [84] proposed a security solution using blockchain to evaluate performance parameters and analyse and detect correlations between traffic functions in smart home networks. Bringhenti et al. [85] presented a configurable automation security system to secure personalization data in smart homes and improve usability by minimizing human interventions and implementing policy-based management. Allifah and Zualkernan [86] presented a novel methodology to rank the security of home consumer devices. They also discussed the analytic hierarchy process (AHP) and applied it to ranking the overall security risks. Thammarat and Techapanupreeda [87] proposed a protocol to fill the gap in security messages in smart homes regarding confidentiality, integrity, and mutual authentication using symmetric cryptography. They demonstrated the efficacy of their protocol using the Burrows–Abadi–Needham (BAN) logic and scyther tool framework.

The security and privacy of smart homes are of concern within the field of cybersecurity. Research on IoT security in smart homes has focused on identifying and addressing issues, such as unauthorized device access, vulnerabilities in PII, cybersecurity vulnerabilities in smart home infrastructure, and potential attacks on authentication and home automation systems. Proposed solutions to these security concerns include using blockchain technology to ensure the integrity of smart home devices and implementing policy-based management to secure personalization data. Additionally, protocols using symmetric cryptography have been suggested to protect confidentiality, integrity, and mutual authentication in smart homes.

### 3.3. IoT Cybersecurity Research

In this section, we summarized the IoT cybersecurity research papers related to smart cities, as shown in Table 3. Abomhara and Køien [88] classified the types of cyberthreats to IoT devices and services and analysed the characteristics of attackers. Rohokale and Prasad [89] proposed an approach for designing robust cybersecurity solutions for IoT device networks since heterogeneous networks are targeted by attackers and often encounter cyberthreats.

Saadeh et al. [90] presented a literature review of authentication and communication processes between IoT objects. Sivanathan et al. [91] evaluated the vulnerabilities of IoT devices to cyberattacks by rating their confidentiality, integrity, availability, and reflectiveness capabilities as either good, average, or poor. The evaluation process was divided into four categories: confidentiality, integrity, availability, and reflectiveness capabilities against attacks.

Neshenko [92] generated cyberthreat intelligence related to Internet-scale inference and the assessment of malicious activity generated by compromised IoT devices for the immediate detection, mitigation, and prevention of IoT exploitation. Ainane et al. [93] described how flooding occurs when data are exchanged between citizens and smart cities. They also identified the types of protocols that revolve around the IoT applications that are used during these exchanges. Vrabie [94] presented IoT services that could help to develop smart cities and provided numerous examples of cities that have implemented these concepts. Viswanadham and Jayavel [95] surveyed related research on blockchain technology implemented within IoT devices and applications. They aimed to provide an understanding of blockchain security and privacy features for IoT services. Lewis [96] used a graph database to understand the complexity of IoT networks, as well as different devices that impact the security of networks and associated data. Wu et al. [97] proposed a framework for the development of future IoT applications and analysed the future directions of IoT communication and its challenging aspects.

In 2019, James [98] attempted to fill the gap in the research on cybersecurity challenges in IoT services and applications by conducting intrusion attacks on several IoT devices within smart homes. They also established a method to protect affected devices and smart home systems from future attacks using an intrusion prevention system. Shokeen et al. [99] suggested a framework to assess the risks of cyberthreats to IoT systems that avoids external factors from being involved in the evaluation. Furthermore, it also reduces existing vulnerabilities. Roukounaki et al. [100] proposed deploying security data collection systems in complex IoT devices and applying effective security analysis algorithms to identify threats, vulnerabilities, and related attack patterns. Van Kleek et al. [101] proposed disaggregating the privacy of IoT networks to help prevent private end-user data from being collected. Thorburn et al. [102] presented possible future development directions for third-party entities that collect personal information without the knowledge of homeowners, based on data flows in smart home environments. Nwafor and Olufowobi [103] presented a framework to detect anomalous system events in IoT ecosystems and associated devices. Ullah et al. [104] proposed a solution to classify the performance of measuring cyberthreats to IoT devices.

In 2020, Sharma et al. [105] discussed the IoT cybersecurity issues and noted that the innovation of IoT devices is growing and expanding progressively. Karie et al. [106] focused on developing combat strategies against IoT cybersecurity threats and also discussed future directions in this domain. Andrade et al. [107] proposed a model for evaluating the risk levels related to IoT cybersecurity. The assessment model was based on a systematic literature review of research on developing smart city applications and cybersecurity risk levels. Singh et al. [108] provided several examples of cyberthreats and countermeasures, as well as discussing how the combination of cloud computing and IoT devices could be used in smart city applications to identify these types of security threats.

In 2021, research on IoT was very active, especially in relation to smart cities. Cvitić et al. [109] presented a model to detect DDoS traffic and identify IoT devices that were categorized into four different classes. Jhanjhi et al. [110] proposed a solution to analyse cybersecurity privacy challenges by understanding the current state of cybersecurity. Ahmed et al. [112] discussed various aspects of cybersecurity in IoT networks and analysed MLP, CNN, LSTP, and AI/ML models. Strecker et al. [111] presented a cyberthreat intelligence model to evaluate and infer malicious activities targeting IoT devices and their data integrity. This model could also mitigate the exploitation of IoT devices. Houichi et al. [113] discovered that ML could be used to detect anomalous threats and vulnerabilities in IoT devices and localize them, as well as generating reports and alerts for the threats. This solution was evaluated experimentally using the NSL-KDD dataset and demonstrated high accuracy (99.31%).

Bhargava et al. [114] suggested enhancing the experience in smart cities by addressing security and privacy issues in IoT platforms and provided an overview of how ML and DL could be implemented in IoT devices and services. Al Solami [115] presented a framework for secure resource administration that enhances IoT services in smart city applications by preventing replication. This involves the distribution of the cloud, networks, IoT platforms, and sensors. They also demonstrated how to prevent unintended attacks from original supplier sources by monitoring non-replicated services. Hulicki and Hulicki [116] explored cyberthreats and vulnerabilities of customer premises networks and the resulting attacks associated with IoT applications and services. Ali et al. [117] discussed the security issues in IoT devices and services that have been gathered and reported. They also classified these issues and provided solutions. Debnath and Chettri [118] reviewed the current trends in IoT research, as well as identifying the recent issues and challenges in IoT applications and industries. Toutsop et al. [119] proved that hackers can exploit sensors and gain unauthorized access to IoT networks. Furthermore, they attempted to carry out DoS attacks on IoT devices to understand the device vulnerabilities and provide intrusion detection using ML and DL. Balaji et al. [120] analysed the types of cyberthreats and consequences that IoT systems may face. They also discussed how to prevent and avoid these attacks.

Khan [121] suggested different privacy protection methods based on pseudonymization, clustering, anonymization, and more to prevent private data from being exchanged with service providers and third parties. Kowta et al. [123] discussed the various cybersecurity threats and vulnerabilities in IoT devices. They performed several attacks on IoT devices and devised with solutions and methods to prevent these attacks. Nakkeeran and Mathi [122] provided a framework for end-to-end IoT sensor and device solutions and a detection method for identifying suspicious and anomalous network behaviour through cross-layer analysis. Maidamwar et al. [124] reviewed the design of an intrusion detection framework for the WSN-based IoT, which they described as being able to increase confidence in the reliability of IoT networks and contain network intrusions. Raimundo and Rosário [125] filled a gap in the research on cybersecurity risks in the IoT domain by discussing the existing solutions and cyberthreats in the industrial Internet of Things (IIoT), based on a literature review. Fan et al. [126] provided general security guidelines for enhancing the IoT in smart cities, which were presented in four main points. Firstly, they provided an overview of recent innovations and common security challenges. Secondly, they discussed the latest security implementations that use cryptography in the IoT. Thirdly, they analysed the security challenges using the activity–network–things architecture. Lastly, they discussed potential IoT security prospects.

Cybersecurity is a critical concern for the widespread adoption of IoT devices and services. To address this challenge, robust cybersecurity solutions have been proposed, including vulnerability assessments for IoT devices, cyberthreat intelligence generation, AI/ML-based threat detection, and models for mitigating malicious activities targeting IoT devices. The combination of cloud computing and IoT devices is also being explored as a means of enhancing the security of smart city applications.

## 4. Cyber Forensics in Smart Cities

Next, we summarized the findings of studies on smart city components from a cyber forensics perspective. Although research on cyber forensics related to IoT devices has been active for some time, progress in research on cyber forensics for smart cities is still slow compared to that on smart city cybersecurity. Most studies related to cyber forensics have focused on smart homes and autonomous vehicles.

### 4.1. Cyber Forensics for the Service and Application Layer

In this section, we summarized the Cyber forensics-related research on each service element within smart cities, as shown in Table 4. Ryu et al. [127] examined certain challenges in digital forensics for smart home systems based on the IoT. The experimental results revealed that cookies could provide information pertaining to user locations. Awasthi et al. [128] conducted forensics research on Almond+, a smart home hub that includes iOS/Android companion apps, a home hub, and a cloud environment. They identified important log locations across the Almond+ and companion applications that could be crucial for investigations. Brotsis et al. [129] presented a blockchain-based solution, cyber-trust blockchain (CTB), that was built on the HyperLedger Fabric. They proposed forensic evidence collection for small offices/home offices in smart home networks using a simulated adversarial model. The authors found the sources of cyberattacks by capturing suspicious network traffic detected by smart home gateway agents (SGPs), inspecting compromised devices, and collecting forensic evidence. This evidence was then securely stored in an off-chain database and hashes and metadata were stored on blockchain to ensure a chain of custody.

Iqbal et al. [130] explored the feasibility of the forensic analysis of different smart plugs brands (D-Link, TP-Link, Telldus, Amazon, and LG) and examined the challenges in resource-constrained devices, such as smart plugs. The authors also reviewed the current related work in the field of the forensic analysis of smart plugs. Kim et al. [131] primarily focused on acquiring, categorizing, and forensically analysing data from Google Nest Hub, Samsung Smart Things, and Kasa cam. The authors examined smart home data collected by companion apps, web interfaces, and APIs. They described how various smart home data could be used as key evidence in certain forensics situations.

Several researchers have also conducted forensics studies on autonomous vehicles to identify artefacts and evidence for investigations. Feng et al. [132] discussed the vulnerabilities in smart autonomous automated vehicles (AAVs) against the backdrop of smart cities and identified potential attack vectors and sources of digital evidence collection. They also proposed a forensics model for vehicle data investigations. Hossain et al. [133] presented a Trust-IoV framework consisting of a forensics gateway and an IoV forensics service to support investigations involving cybercrime cases in IoV environments. They also proposed a model to collect evidence from distributed IoV frameworks.

In 2022, Zhang et al. [134] implemented a lightweight incentive authentication system (LIAS) developed using a three-tier architecture, including a user layer, fog layer, and cloud layer. They used the fog-assisted IoV with the pairing-free certificateless signcryption to create an anonymous authentication system. A pseudonym update mechanism was used to defend against DDoS attacks and provide a fair mechanism to allow vehicles to join the forensics services. Vehicles with cameras could record pictures of roadside infrastructure and send them to the cloud anonymously. These stored images could then be valuable evidence in future investigations. Tyagi et al. [135] proposed an AI-enabled blockchain solution that was implemented on a local Ethereum blockchain platform as a proof of concept for intelligent digital forensics for automated connected vehicles (ACVs) in smart cities. Short random signatures could be used to anonymously authenticate witness identities, privacy, and trust.

### 4.2. IoT Cyber Forensics Research

In this section, we summarized the IoT Cyber forensics-related research on smart cities, as shown in Table 5. Zia et al. [136] proposed an application-specific forensics model for the IoT that included the collection, examination, analysis, and reporting of IoT devices in smart environments. They also identified potential sources of digital evidence and artefacts from smart homes (Nest smart), smart cities with intelligent traffic management system (ITMSs), wearable devices (VitalPatch), and network and cloud forensics. Rizal et al. [137] presented a network forensics model for detecting flooding attacks on IoT devices. They used a Bluetooth Arduino device to simulate flooding attacks and then monitored and stored the logs of abnormal activity on the infected Arduino device. These logs were further analysed using packet monitoring tools, such as Wireshark, to identify malicious IPs.

In 2019, Hou et al. [138] presented a systematic review of the impacts of the IoT on digital forensics. They reviewed 58 papers that were published from 2010 to 2018. They then presented the landscape of the IoT in three different dimensions: spatial, temporal, and technical. The spatial dimension focuses on the potential sources of evidence, the temporal dimension focuses on the legal acceptance of evidence, and the technical dimension focuses on the needed tools and technologies for data collection and analysis. Jayakrishnan and Vasanthi [139] presented an analysis of IoT attacks on Wi-Fi cameras and side-channel attacks (SCAs) to obtain AES keys to aid investigators in understanding attack patterns. Qatawneh et al. [140] proposed a new digital forensics investigation model (DFIM) for the IoT. They categorized three zones (cloud, fog, and precipitation) as the potential sources of IoT digital evidence. The model consists of a data provider zone (DPZ), which is responsible for assigning all data gathered by sensor nodes into groups and assigning investigators to the case. Yaqoob et al. [141] examined novel IoT issues within conventional computer forensics. They also analysed the advantages and disadvantages of recent studies on IoT forensics by creating a taxonomy based on forensic stages.

**Figure 4:**
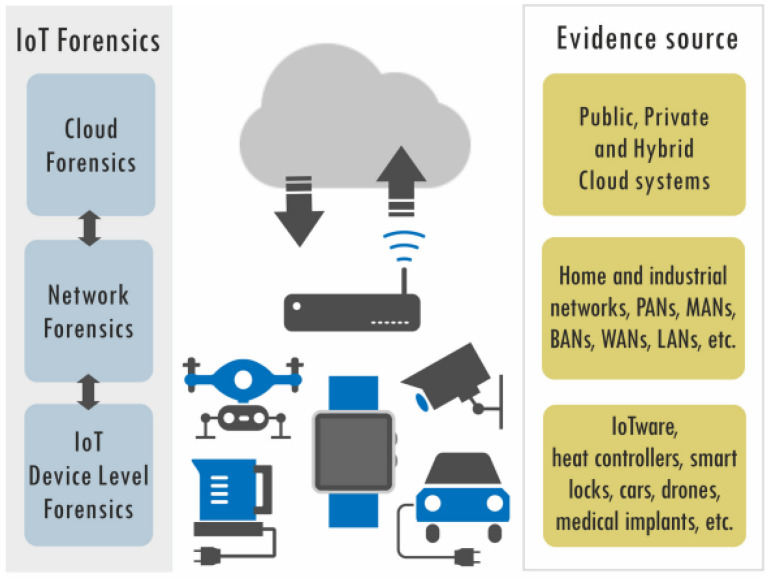
The components of IoT forensics [142].

Numerous studies related to IoT forensics were published in 2020. In the same year, Stoyanova et al. [142] identified the main issues involved in IoT investigations, including legal challenges, privacy concerns, and cloud security challenges, as illustrated in Figure 4. They proposed decentralized blockchain-based solutions to secure digital evidence obtained from IoT devices. They also created the IoT challenge mind map and IoT attack taxonomy. In addition, Patil et al. [143] presented a methodology for cyber forensics in IoT architectures, which includes a sensing layer, network layer, service layer, and interface layer. They conducted a study on events in wireless cyber–physical systems (WCPs), revealing API calls to RESTFul web services from an Amazon Echo. They also performed a comparative analysis of the existing research on IoT forensics. Jayakrishnan and Vasanthi [144] presented the HoneyNetCloud investigation model (HIM), which uses honeypots to record attacker behaviour. They captured IoT network attack data for one year and classified them using the Dempster–Shafer theory (DST). The proposed model was designed to assist investigators in conducting investigations on IoT network attacks.

Atlam et al. [145] reviewed IoT forensics by emphasizing the need for AI in this field. They discussed IoT security challenges within digital forensics and the investigation processes for IoT forensics. Patel and Malek [146] discussed existing forensics frameworks, security aspects, and tools for IoT forensics. They also covered some of the open issues in IoT forensics, such as device storage limits, heterogeneous ecosystems, and cloud forensics. Yang et al. [147] introduced biometric-based IoT authentication in two stages: the enrolment stage and authentication stage, including a feature extraction module. They classified IoT forensics into three different layers (device forensics, network forensics, and cloud forensics) and analysed recent advances in IoT forensics. Bandil and Al-Masri [148] found abnormalities in real-time events connected to IoT data streams. They suggested VTA-IH, a fog-based digital forensics platform that uses the complex events processing (CEP) concept.

In 2021, Janarthanan et al. [149] reviewed the solutions and frameworks related to IoT forensics that were presented in recent studies. They analysed various digital forensics frameworks specific to the IoT, based on the forensic process stages, and evaluated their strengths and limitations. Surange and Khatri [150] presented a comparison of IoT frameworks, with a focus on the level of forensics in the IoT at the device, cloud, fog/edge, cloud/fog, and device/network levels. They also classified IoT challenges based on forensic processing, investigation, and IoT architectures. Kim et al. [151] demonstrated a data acquisition framework for wearable devices from Xiaomi (Amazft Stratos 3 and Mi Band4), Huawei, LG, and Fitbit. They analysed the devices using ADB to extract artefacts pertaining to health and device information. Ganesh et al. [152] investigated AI use cases in forensics in different areas, such as blockchain, the IoT, and cloud computing, and examined forensic application cases in these areas using AI. They also conducted a systematic literature review to achieve the study’s objective.

Some studies have also been conducted across multiple domains. Sharma and Singh [153] discussed the deep packet inspection (DPI)-based forensic analysis of network traffic, along with the use of grid infrastructure to lower the computational costs of processing packet captures in real time. Mishra et al. [154] demonstrated the use of various open-source network analysis tools and presented various packet capture and aggregating information from different sources in different environments, such as honeypots and honeynets, virtual honeypots, and parallel and serial architectures.

## 5. Future Expectations and Research Directions

Based on our literature review, the IoT was identified as the most critical technological component of smart cities. Consequently, numerous researchers have been conducting research on IoT cybersecurity. Previous studies have focused on DoS attacks, authentication, and the control of access to IoT devices. Furthermore, various frameworks for defending IoT devices against attacks have been proposed and research on intrusion detection and prevention systems is also ongoing. With the recent technological advancements, research on the use of ML/DL for IoT devices is also progressing.

Smart homes consist of smart hubs and other devices that are connected either via Zigbee, Z-Wave, or IoT communication protocols. As previous studies have suggested, digital forensics in smart homes is vital to reconstruct past events during forensic investigations. Relevant artefacts collected from smart home devices can help to identify the location of devices connected to the smart homes. Traces from these devices can be found in different data types, such as XML, protobuf, JSON, DB, and archive logs, which can coexist on devices or smartphone applications. Forensic investigators must familiarize themselves with device-level knowledge, communication protocols, and data types before attempting to investigate such events.

The emergence of autonomous and semi-autonomous vehicles in smart cities is gaining popularity due to their relation to the highly connected infrastructure that is a perfect enabler of such vehicles. The control systems at the hearts of these vehicles collect data from sensors for collision warning systems (CWSs) and collision avoidance systems (CASs) to prevent accidents, often involving communication with smart city ITS. However, the susceptibility of AVs to cyberattacks cannot be ignored due to their connectivity with other IoT devices. Previous studies have suggested a few preventive measures to defend against such cyberattacks, as well as the use of Ethereum blockchain as a platform for AV digital forensics. Recent research on this new technology has not only helped to identify potential threats but has also guided investigators when examining AV incidents, such as crash investigations and security and privacy breaches.

The potential of the IoT to meet the needs of smart cities is immense; however, the heterogeneity of IoT devices, embedded software, and hardware, coupled with their limited local data processing capabilities, presents new challenges for forensic investigators. According to some studies, digital forensics for the IoT should be tailored to specific applications within smart cities, while traditional digital forensics should be employed for smart homes. These studies have also suggested identifying potential data of interest in the IoT applications within smart cities. The integration of edge and fog computing in IoT ecosystems is on the rise due to its interoperability and increased efficiency. Meanwhile, although theoretical and conceptual IoT forensics frameworks for the device, cloud, fog, and edge levels exist, practical solutions are still lacking.

In addition to the research areas mentioned, there are other emerging research areas in the smart city field that are attracting attention. For instance, the integration of blockchain technology into smart cities is a growing area of interest due to its potential for secure and transparent transactions. Blockchain technology can be used to secure IoT devices and protect the privacy of citizens living in smart cities. Furthermore, the use of drones for various purposes, such as monitoring and delivering goods, is another emerging research area.

## 6. Summary and Conclusions

A thorough review of research on cybersecurity and cyber forensics in smart cities, which included papers that were published from 2015 to 2022, was conducted. Smart transportation, smart communities, smart living, and smart environments were identified as the major components of smart cities. Additionally, the service and application layer, technical layer, network communication layer, and device layer were recognized as the different layers of smart cities.

Through our review of cybersecurity and cyber forensics studies, we discovered that research related to smart homes and smart mobility was the most active. Moreover, many studies have been conducted on IoT devices from a technical point of view. Compared to cybersecurity, research on cyber forensics in smart cities is relatively scarce, despite the fact that smart cities promote urban regeneration by creating more inclusive, safe, and sustainable cities. Research on IoT devices is still being carried out but not many studies have been conducted specifically on smart cities, highlighting the lack of research on cyber forensics.

Since 2020, numerous studies based on ML and DL have been conducted on the IoT, which is an important technological element of smart cities. Because large-scale data are transmitted through IoT devices in smart cities, ML and DL remain essential factors in smart city research. Moreover, because cybercrime that affects everyday life in smart cities has become more complicated, research on cyber forensics in related devices is of great importance.

Another important aspect to consider is the ethical implications of cybersecurity and cyber forensics in smart cities. Smart cities collect vast amounts of data from citizens, raising concerns about privacy and security. Researchers need to address the ethical concerns related to data collection, storage, and analysis in smart cities.

Overall, the fields of cybersecurity and cyber forensics in smart cities are rapidly evolving and present numerous research opportunities. This review article aimed to assist scholars who are interested in cybersecurity and cyber forensics for smart cities in gaining a better understanding of their current status and possible future directions. In this way, other researchers can continue to contribute toward building more secure, inclusive, and sustainable smart cities.

## Figures and Tables

**Table 1:** The key elements of smart cities derived from the literature.

Ref.	Key Elements
Khan et al. [15]	Smart homes, smart education, smart governance, smart environments, smart communities, smart healthcare, smart transportation, and smart energy
Fournaris et al. [16]	Application (traffic management, waste management, smart parking, smart buildings, public safety, etc.), processing (service platforms, APIs, data storage, information processing, network management, etc.), communication (wireless networks (Wi-Fi, 4G, LoRa, ZigBee, and NB-IoT), optical networks, telephony, etc.), and sensing (sensors (temperature, traffic, parking, etc.), citizens (mobile phones, etc.), radio frequency identification (RFID), cameras, etc.)
Sinaeepourfard et al. [17]	Smart city applications (situation rooms, control centers, and applications), city operating systems (city semantics, analytics, processes, events repository and management, and security management), and city information sources (sensors, cameras, and apps)
Kim et al. [18]	Smart homes, smart mobility, smart people, and smart economy
Hu [19]	Public services, public security, urban governance, smart industries, smart city operation and management centers, urban big data centers, and smart city sensor network systems
Moustaka et al. [20]	Smart living, smart mobility, smart people, smart environments, smart economy, and smart governance
Fraga-Lamas et al. [21]	Smart energy, smart industries, smart networks, defense and public safety, smart living, smart retail, smart healthcare, smart farming, and smart transportation

**Table 2:** Cybersecurity for the service and application layer of smart cities.

Category	Authors	Year	Approach/Experiment
Smart Mobility	Javed et al. [63]	2016	ECC-based digital signatures and the encryption of smart city ITSs
	Wang et al. [64]	2019	Key protection strategies for smart vehicles
	Kim et al. [9]	2021	A systemic survey of research on autonomous attacks and defence
	Sharmila et al. [65]	2022	A mitigation model of cybersecurity vulnerabilities and threats
	Chen and Quan [66]	2022	Security solutions for the BIoV and its security challenges
Smart Homes	Ryu and Kwak [67]	2015	Authenticated devices and safe access controls for smart homes
	McGee [68]	2016	The development of SECaaS to evaluate smart home ecosystem data-link layers
	Liu and Hu [69]	2016	Algorithms to prevent hackers from altering energy bills
	Nsunza et al. [70]	2017	An evaluation of the performance of TCP and UDP network traffic by FPGA
	Gamundani et al. [71]	2018	Cyberattack and authentication threats on the IoT in smart homes
	Ghirardello et al. [72]	2018	An analysis of various attacks and key vulnerabilities
	Kraemer and Flechais [73]	2018	A systematic review of research on privacy in smart homes
	Bastos et al. [74]	2018	IoT security solutions and anticipated attacks on data protocols
	Sturgess et al. [75]	2018	A discussion of heterogeneous threats to personal information
	Siddhanti et al. [76]	2019	Good practice in cybersecurity maturity assessment
	Elmisery and Sertovic [77]	2020	The preservation of event log privacy for smart homeowners
	Rossi et al. [78]	2020	Monitoring and defence against exploiting devices using Shodan APIs
	Giannoutakis et al. [79]	2020	Blocking malicious IPs using dynamic and immutable management
	Rauti et al. [80]	2021	A demonstration of a man-in-the-browser attack on a smart home system
	Awang et al. [81]	2021	Solutions for threats to smart home operational environments
	Turner et al. [82]	2021	Best practice and recommendations for smart homeowners
	Alshboul et al. [83]	2021	Protecting sensor identities from being recognized
	Mahor et al. [84]	2022	A multivariate correlation analysis and correlation detection
	Bringhenti et al. [85]	2022	Cybersecurity personalization based on policy-based management
	Allifah and Zualkernan [86]	2022	A ranking of the critical vulnerabilities of smart home devices
	Thammarat and Techapanupreeda [87]	2022	A symmetric cryptographic protocol for smart homes

**Table 3:** A summary of the approaches and experiments presented in existing IoT cybersecurity research.

Category	Authors	Year	Approach/Experiment
IoT	Abomhara and Køien [88]	2015	Threat classification, attack characterization and an analysis of IoT devices
	Rohokale and Prasad [89]	2015	A critical systematic review of cyberthreats to heterogeneous IoT networks
	Saadeh et al. [90]	2016	A survey of IoT authentication techniques
	Sivanathan et al. [91]	2017	An evaluation of vulnerabilities through CIA and the capability of DDoS attacks
	Neshenko [92]	2018	An evaluation of large-scale malicious IoT data from cyberthreat intelligence
	Ainane et al. [93]	2018	An identification of data and protocol exchanges between users and IoT applications
	Vrabie [94]	2018	How IoT networks and services can develop smart cities
	Viswanadham and Jayavel [95]	2018	The implementation of blockchain in IoT security and its domain
	Lewis [96]	2018	The security of IoT networks and devices
	Wu et al. [97]	2018	Hyperconnected interdependent IoT services and future applications
	James [98]	2019	The detection of critical attacks on IoT devices by executing cybersecurity- based attacks
	Shokeen et al. [99]	2019	An accurate evaluation of each vulnerability of IoT systems
	Roukounaki et al. [100]	2019	The identification of threats using security analytics algorithms, vulnerabilities, and attacks
	Van Kleek et al. [101]	2019	The classification of IoT device data in privacy-empowering networks
	Thorburn et al. [102]	2019	Basic future frameworks for IoT devices that share data with third parties
	Nwafor and Olufowobi [103]	2019	An anomaly detection system for events in IoT ecosystems
	Ullah et al. [104]	2019	The detection of infected SW and files across IoT networks using deep learning (DL)
	Sharma et al. [105]	2020	The computerized security of IoT devices
	Karie et al. [106]	2020	A literature review of IoT threat detection and security challenges
	Andrade et al. [107]	2020	An evaluation of IoT cybersecurity maturity in smart cities according to its risk levels
	Singh et al. [108]	2020	The types of IoT security threats and their countermeasures
	Cvitić et al. [109]	2021	The detection of DDoS traffic attack using logistic model trees on different IoT devices
	Jhanjhi et al. [110]	2021	An investigation of cyberattacks that target the four layers of the IoT
	Strecker et al. [111]	2021	The detection of malicious and anomalous data in IoT systems using machine learning (ML)
	Ahmed et al. [112]	2021	An analysis of the security and privacy concepts in IoT networks using ML and DL
	Houichi et al. [113]	2021	The detection of infected IoT devices, alerts, and reports based on ML and other methods
	Bhargava et al. [114]	2021	The implementation of ML/DL on IoT platforms to prevent security issues
	Al Solami [115]	2021	The use of secure resource administration to stop IoT services from being replicated
	Hulicki and Hulicki [116]	2021	The threats to network security mechanisms and their vulnerabilities to attacks
	Ali et al. [117]	2021	A literature review of IoT security issues, their classification, and solutions
	Debnath and Chettri [118]	2021	A literature review of the security challenges in IoT applications
	Toutsop et al. [119]	2021	DoS attacks on IoT devices through networks
	Balaji et al. [120]	2021	An analysis of cyberthreats and prevention methods for avoiding IoT attacks
	Khan [121]	2021	Privacy preservation based on pseudonymization and anonymization
	Nakkeeran and Mathi [122]	2021	The detection of anomalies in IoT networks and solutions for cross-layer issues
	Kowta et al. [123]	2022	A identification of security threats to different IoT devices by implementing attacks on them
	Maidamwar et al. [124]	2022	An intrusion detection design for the WSN-based IoT
	Raimundo and Rosário [125]	2022	A literature review of cybersecurity threats to the IoT
	Fan et al. [126]	2022	Security guidelines for developing IoT-enabled smart cities

**Table 4:** A summary of the research on cyber forensics for smart homes and autonomous vehicles.

Category	Authors	Year	Approach/Experiment
Smart Homes	Ryu et al. [127]	2017	Smart home forensics models based on attack scenarios
	Awasthi et al. [128]	2018	Forensic data acquisition and analysis based on the smart home Wi-Fi system Almond+
	Brotsis et al. [129]	2019	Smart home data collection and the preservation of evidence using blockchain
	Iqbal et al. [130]	2020	Challenges in smart plug forensic analysis and investigation
	Kim et al. [131]	2020	Google Nest Hub, Samsung Smart Things, and Kasa Cam forensics
Autonomous Vehicles	Feng et al. [132]	2017	Digital forensics models for autonomous vehicle cases
	Hossain et al. [133]	2017	Trusting the IoV to collect and store evidence from distributed infrastructure
	Zhang et al. [134]	2022	An incentive lightweight authentication scheme for forensics services in the IoV
	Tyagi et al. [135]	2022	Using local Ethereum blockchain to collect evidence from connected vehicles

**Table 5:** A summary of the approaches and experiments presented in existing IoT and multi-area research.

Category	Authors	Year	Approach/Experiment
IoT	Zia et al. [136]	2017	An application-specific digital forensics investigative model for the IoT
	Rizal et al. [137]	2018	An evaluated network forensics method to detect flooding attacks
	Hou et al. [138]	2019	A survey of IoT forensics in the technical, temporal, and spatial dimensions
	Jayakrishnan and Vasanthi [139]	2019	An attack simulation to identify advanced encryption standard (AES) keys
	Qatawneh et al. [140]	2019	A digital forensics investigation model (DFIM) for the IoT
	Yaqoob et al. [141]	2019	An IoT taxonomy based on different forensics processes
	Stoyanova et al. [142]	2020	Cloud security challenges and data acquisition using blockchain
	Patil et al. [143]	2020	A comparative analysis of IoT cyber forensics research
	Jayakrishnan and Vasanthi [144]	2020	A process model for forensics using an IoT HoneyNetCloud
	Atlam et al. [145]	2020	IoT forensics techniques and the need for AI in IoT forensics
	Patel and Malek [146]	2020	Existing IoT forensics frameworks and their challenges
	Yang et al. [147]	2020	Biometric-based authentication and forensics for the IoT
	Bandil and Al-Masri [148]	2020	Real-time events associated with IoT data streams
	Janarthanan et al. [149]	2021	Challenges in smart home investigations and forensics
	Surange and Khatri [150]	2021	A survey of developments in IoT forensics and the identification of research gaps
	Kim et al. [151]	2022	A data acquisition framework for smart devices
	Ganesh et al. [152]	2022	AI use cases of forensics in blockchain, the IoT, and cloud computing
Multi- Area	Sharma and Singh [153]	2015	A forensic analysis of deep packet inspection (DPI) networks
	Mishra et al. [154]	2021	The identification of suspicious packets to detect cybercrimes using a packet analyser

## Data Availability

Not applicable.

## References

[1] Alotaibi A., Alsubaie D., Alaskar H., Alhumaid L., Thuwayni R.B., Alkhalifah R., Alhumoud S. Kingdom of Saudi Arabia: Era of Smart Cities. Proceedings of the 2022 2nd International Conference on Computing and Information Technology (ICCIT).

[2] Shamsuzzoha A., Nieminen J., Piya S., Rutledge K. (2021). Smart city for sustainable environment: A comparison of participatory strategies from Helsinki, Singapore and London. Cities.

[3] Farag A.A. (2019). The story of NEOM city: Opportunities and challenges. New Cities and Community Extensions in Egypt and the Middle East.

[4] (2021). Smart Sustainable Cities. https://www.itu.int/en/mediacentre/backgrounders/Pages/smart-sustainable-cities.aspx.

[5] Cavada M., Tight M.R., Rogers C.D. (2019). A smart city case study of Singapore—Is Singapore truly smart?. Smart City Emergence.

[6] Willems J., Bergh J.V.d., Viaene S. (2017). Smart city projects and citizen participation: The case of London. Public Sector Management in a Globalized World.

[7] Gon K.K., Hoon K.S. Using Threat Modeling for Risk Analysis of SmartHome. Proceedings of the Symposium of the Korean Institute of Communications and Information Sciences.

[8] Kim K., Cho K., Lim J., Jung Y.H., Sung M.S., Kim S.B., Kim H.K. (2020). What is your protocol: Vulnerabilities and security threats related to Z-Wave protocol. Pervasive Mob. Comput..

[9] Kim K., Kim J.S., Jeong S., Park J.H., Kim H.K. (2021). Cybersecurity for autonomous vehicles: Review of attacks and defense. Comput. Secur..

[10] Miller C., Valasek C. (2015). Remote exploitation of an unaltered passenger vehicle. Black Hat USA.

[11] Ji-Young K., In L.J., Gon K.K. The all-purpose sword: North korea’s cyber operations and strategies. Proceedings of the 2019 11th International Conference on Cyber Conflict (CyCon).

[12] Lee S., Kim H.K., Kim K. (2019). Ransomware protection using the moving target defense perspective. Comput. Electr. Eng..

[13] Losavio M.M., Chow K., Koltay A., James J. (2018). The Internet of Things and the Smart City: Legal challenges with digital forensics, privacy, and security. Secur. Priv..

[14] Baig Z.A., Szewczyk P., Valli C., Rabadia P., Hannay P., Chernyshev M., Johnstone M., Kerai P., Ibrahim A., Sansurooah K. (2017). Future challenges for smart cities: Cyber-security and digital forensics. Digit. Investig..

[15] Khan S.M., Chowdhury M., Morris E.A., Deka L. (2019). Synergizing Roadway Infrastructure Investment with Digital Infrastructure: Motivations, Current Status and Future Direction. ASCE J. Infrastruct. Syst.

[16] Fournaris A.P., Lampropoulos K., Koufopavlou O. End Node Security and Trust vulnerabilities in the Smart City Infrastructure. Proceedings of the MATEC Web of Conferences.

[17] Sinaeepourfard A., Garcia J., Masip-Bruin X., Marín-Tordera E., Cirera J., Grau G., Casaus F. Estimating Smart City sensors data generation. Proceedings of the 2016 Mediterranean Ad Hoc Networking Workshop (Med-Hoc-Net).

[18] Kim K., Alfouzan F.A., Kim H. (2021). Cyber-Attack Scoring Model Based on the Offensive Cybersecurity Framework. Appl. Sci..

[19] Hu R. (2019). The state of smart cities in China: The case of Shenzhen. Energies.

[20] Moustaka V., Vakali A., Anthopoulos L.G. (2018). A systematic review for smart city data analytics. ACM Comput. Surv. (cSuR).

[21] Fraga-Lamas P., Fernández-Caramés T.M., Suárez-Albela M., Castedo L., González-López M. (2016). A review on internet of things for defense and public safety. Sensors.

[22] https://www.flaticon.com.

[23] Serrano M., Griffor E., Wollman D., Dunaway M., Burns M., Rhee S., Greer C. (2022). Smart Cities and Communities: A Key Performance Indicators Framework.

[24] Kumar T.M.V. (2020). Smart Environment for Smart Cities. Advances in 21st Century Human Settlements.

[25] Oh I.K., Seo J.W., Lee M.K., Lee T.H., Han Y.N., Park U.S., Ji H.B., Lee J.H., Cho K.H., Kim K. (2020). Derivation of Security Requirements of Smart TV Based on STRIDE Threat Modeling. J. Korea Inst. Inf. Secur. Cryptol..

[26] Yamin M.M., Shalaginov A., Katt B. Smart policing for a smart world opportunities, challenges and way forward. Proceedings of the Future of Information and Communication Conference.

[27] Navarathn P.J., Malagi V.P. Artificial Intelligence in Smart City Analysis. Proceedings of the 2018 International Conference on Smart Systems and Inventive Technology (ICSSIT).

[28] Talari S., Shafie-Khah M., Siano P., Loia V., Tommasetti A., Catalão J.P. (2017). A review of smart cities based on the internet of things concept. Energies.

[29] Bauer M., Sanchez L., Song J. (2021). IoT-enabled smart cities: Evolution and outlook. Sensors.

[30] Toma C., Alexandru A., Popa M., Zamfiroiu A. (2019). IoT solution for smart cities’ pollution monitoring and the security challenges. Sensors.

[31] Thirumalaisamy M., Basheer S., Selvarajan S., Althubiti S.A., Alenezi F., Srivastava G., Lin J.C.W. (2022). Interaction of secure cloud network and crowd computing for smart city data obfuscation. Sensors.

[32] Alam T. (2021). Cloud-based IoT applications and their roles in smart cities. Smart Cities.

[33] Park J., Chung H., DeFranco J.F. (2022). Multilayered Diagnostics for Smart Cities. Computer.

[34] Lee I., Park D., Son Y., Lee Y., Park T. (2018). Technology Trends of IoT-based Smart city application. J. Comput. Sci. Eng..

[35] Kumar S., Rathore R.S., Mahmud M., Kaiwartya O., Lloret J. (2022). BEST—Blockchain-Enabled Secure and Trusted Public Emergency Services for Smart Cities Environment. Sensors.

[36] Asif M., Aziz Z., Bin Ahmad M., Khalid A., Waris H.A., Gilani A. (2022). Blockchain-Based Authentication and Trust Management Mechanism for Smart Cities. Sensors.

[37] Ibba S., Pinna A., Seu M., Pani F.E. Citysense: Blockchain-oriented smart cities. Proceedings of the the XP2017 Scientific Workshops.

[38] Alzahrani N.M., Alfouzan F.A. (2022). Augmented reality (AR) and cyber-security for smart cities—A systematic literature review. Sensors.

[39] Jawhar I., Mohamed N., Al-Jaroodi J. (2018). Networking architectures and protocols for smart city systems. J. Internet Serv. Appl..

[40] Elsaeidy A., Elgendi I., Munasinghe K.S., Sharma D., Jamalipour A. A smart city cyber security platform for narrowband networks. Proceedings of the 2017 27th International Telecommunication Networks and Applications Conference (ITNAC).

[41] Xu K., Wan Y., Xue G. (2019). Powering smart homes with information-centric networking. IEEE Commun. Mag..

[42] Garcia-Font V., Garrigues C., Rifà-Pous H. (2016). A comparative study of anomaly detection techniques for smart city wireless sensor networks. Sensors.

[43] Garcia-Font V., Garrigues C., Rifà-Pous H. (2017). Attack classification schema for smart city WSNs. Sensors.

[44] Alfouzan F.A., Kim K., Alzahrani N.M. (2022). An efficient framework for securing the smart city communication networks. Sensors.

[45] Biswas K., Muthukkumarasamy V. Securing smart cities using blockchain technology. Proceedings of the 2016 IEEE 18th International Conference on High Performance Computing and Communications, IEEE 14th International Conference on Smart City, IEEE 2nd International Conference on Data Science and Systems (HPCC/SmartCity/DSS).

[46] Jiang J.C., Kantarci B., Oktug S., Soyata T. (2020). Federated learning in smart city sensing: Challenges and opportunities. Sensors.

[47] Kalinin M., Krundyshev V., Zegzhda P. (2021). Cybersecurity risk assessment in smart city infrastructures. Machines.

[48] AlDairi A. (2017). Cyber security attacks on smart cities and associated mobile technologies. Procedia Comput. Sci..

[49] Neshenko N. (2021). Illuminating Cyber Threats for Smart Cities: A Data-Driven Approach for Cyber Attack Detection with Visual Capabilities. Ph.D. Thesis.

[50] Alassad M., Spann B., Al-khateeb S., Agarwal N. Using computational social science techniques to identify coordinated cyber threats to smart city networks. Proceedings of the Joint International Conference on Design and Construction of Smart City Components.

[51] Hamid S., Bawany N.Z. ACIDS: A Secure Smart City Framework and Threat Model. Proceedings of the 4th International Conference on Wireless, Intelligent and Distributed Environment for Communication.

[52] Alibasic A., Al Junaibi R., Aung Z., Woon W.L., Omar M.A. Cybersecurity for smart cities: A brief review. Proceedings of the Data Analytics for Renewable Energy Integration: 4th ECML PKDD Workshop, DARE 2016.

[53] Al-Turjman F., Zahmatkesh H., Shahroze R. (2022). An overview of security and privacy in smart cities’ IoT communications. Trans. Emerg. Telecommun. Technol..

[54] Alamer M., Almaiah M.A. Cybersecurity in Smart City: A systematic mapping study. Proceedings of the 2021 International Conference on Information Technology (ICIT).

[55] Duan L., Lou Y., Wang S., Gao W., Rui Y. (2018). AI-oriented large-scale video management for smart city: Technologies, standards, and beyond. IEEE MultiMedia.

[56] Lakhouil M., Abtoy A. Cybersecurity of smart cities: A glimpse. Proceedings of the 4th Smart Cities Symposium (SCS 2021).

[57] Ma C. (2021). Smart city and cyber-security; technologies used, leading challenges and future recommendations. Energy Rep..

[58] Figueiredo B.J., Costa R.L.d.C., Santos L., Rabadão C. (2022). Cybersecurity and Privacy in Smart Cities for Citizen Welfare. Smart Cities, Citizen Welfare, and the Implementation of Sustainable Development Goals.

[59] Wang P., Ali A., Kelly W. Data security and threat modeling for smart city infrastructure. Proceedings of the 2015 International Conference on Cyber Security of Smart Cities, Industrial Control System and Communications (SSIC).

[60] Butt T.A., Afzaal M. (2017). Security and privacy in smart cities: Issues and current solutions. Smart Technologies and Innovation for a Sustainable Future: Proceedings of the 1st American University in the Emirates International Research Conference, Dubai, United Arab Emirates 2017.

[61] Andrade R.O., Tello-Oquendo L., Ortiz I. (2021). Cybersecurity Risks of IoT on Smart Cities. Cybersecurity Risk of IoT on Smart Cities.

[62] Juma M., Shaalan K. (2020). Cyberphysical systems in the smart city: Challenges and future trends for strategic research. Swarm Intelligence for Resource Management in Internet of Things.

[63] Javed M.A., Ben Hamida E., Znaidi W. (2016). Security in intelligent transport systems for smart cities: From theory to practice. Sensors.

[64] Wang Z., Wang Y., Zhang Y., Liu Y., Ma C., Wang H. A brief survey on cyber security attack entrances and protection strategies of intelligent connected vehicle. Proceedings of the Smart Computing and Communication: 4th International Conference, SmartCom 2019.

[65] Sharmila V.C., Aslam H.M., Riswan M.M. Analysing and Identifying Harm Propagation of Cyber Threats in Autonomous Vehicles and Mitigation Through ANN. Proceedings of the Smart Trends in Computing and Communications: Proceedings of SmartCom 2021, Las Vegas, NA, USA, 2–3 March 2021.

[66] Chen C., Quan S. (2022). A Summary of Security Techniques-Based Blockchain in IoV. Secur. Commun. Netw..

[67] Ryu H.S., Kwak J. (2015). Secure data access control scheme for smart home. Advances in Computer Science and Ubiquitous Computing: CSA &CUTE.

[68] McGee T.M. (2016). Evaluating the Cyber Security in the Internet of Things: Smart Home Vulnerabilities. Ph.D. Thesis.

[69] Liu Y., Hu S. (2016). Smart home scheduling and cybersecurity: Fundamentals. Smart Cities and Homes.

[70] Nsunza W.W., Rutunda S., Hei X. Design and implementation of a low-cost software defined wireless network testbed for smart home. Proceedings of the Security, Privacy, and Anonymity in Computation, Communication, and Storage: SpaCCS 2017 International Workshops.

[71] Gamundani A.M., Phillips A., Muyingi H.N. An overview of potential authentication threats and attacks on Internet of Things (IoT): A focus on Smart home applications. Proceedings of the 2018 IEEE International Conference on Internet of Things (iThings) and IEEE Green Computing and Communications (GreenCom) and IEEE Cyber, Physical and Social Computing (CPSCom) and IEEE Smart Data (SmartData).

[72] Ghirardello K., Maple C., Ng D., Kearney P. Cyber security of smart homes: Development of a reference architecture for attack surface analysis. Proceedings of the Living in the Internet of Things: Cybersecurity of the IoT-2018.

[73] Kraemer M.J., Flechais I. Researching privacy in smart homes: A roadmap of future directions and research methods. Proceedings of the Living in the Internet of Things: Cybersecurity of the IoT-2018.

[74] Bastos D., Shackleton M., El-Moussa F. Internet of things: A survey of technologies and security risks in smart home and city environments. Proceedings of the Living in the Internet of Things: Cybersecurity of the IoT-2018.

[75] Sturgess J., Nurse J.R., Zhao J. A capability-oriented approach to assessing privacy risk in smart home ecosystems. Proceedings of the Living in the Internet of Things: Cybersecurity of the IoT-2018.

[76] Siddhanti P., Asprion P.M., Schneider B. Cybersecurity by Design for Smart Home Environments. Proceedings of the ICEIS (1).

[77] Elmisery A.M., Sertovic M. Privacy preserving threat hunting in smart home environments. Proceedings of the Advances in Cyber Security: First International Conference, ACeS 2019.

[78] Rossi M.T., Greca R., Iovino L., Giacinto G., Bertoli A. Defensive Programming for Smart Home Cybersecurity. Proceedings of the 2020 IEEE European Symposium on Security and Privacy Workshops (EuroS&PW).

[79] Giannoutakis K.M., Spathoulas G., Filelis-Papadopoulos C.K., Collen A., Anagnostopoulos M., Votis K., Nijdam N.A. A blockchain solution for enhancing cybersecurity defence of IoT. Proceedings of the 2020 IEEE International Conference on Blockchain (Blockchain).

[80] Rauti S., Laato S., Pitkämäki T. Man-in-the-Browser Attacks Against IoT Devices: A Study of Smart Homes. Proceedings of the 12th International Conference on Soft Computing and Pattern Recognition (SoCPaR 2020) 12.

[81] Awang N.F., Zainudin A.F.I.M., Marzuki S., Alsagoff S.N., Tajuddin T., Jarno A.D. (2021). Security and threats in the internet of things based smart home. Innovative Systems for Intelligent Health Informatics: Data Science, Health Informatics, Intelligent Systems, Smart Computing.

[82] Turner S., Nurse J., Li S. When googling it does not work: The challenge of finding security advice for smart home devices. Proceedings of the Human Aspects of Information Security and Assurance: 15th IFIP WG 11.12 International Symposium, HAISA 2021.

[83] Alshboul Y., Bsoul A.A.R., Al Zamil M., Samarah S. (2021). Cybersecurity of smart home systems: Sensor identity protection. J. Netw. Syst. Manag..

[84] Mahor V., Badodia S.K., Kumar A., Bijrothiya S., Temurnikar A. (2022). Cyber Security for Secured Smart Home Applications Using Internet of Things, Dark Web, and Blockchain Technology in the Future. Dark Web Pattern Recognition and Crime Analysis Using Machine Intelligence.

[85] Bringhenti D., Valenza F., Basile C. (2022). Toward Cybersecurity Personalization in Smart Homes. IEEE Secur. Priv..

[86] Allifah N.M., Zualkernan I.A. (2022). Ranking Security of IoT-Based Smart Home Consumer Devices. IEEE Access.

[87] Thammarat C., Techapanupreeda C. Secure Key Establishment Protocol for Smart Homes Based on Symmetric Cryptography. Proceedings of the 2022 International Conference on Information Networking (ICOIN).

[88] Abomhara M., Køien G.M. (2015). Cyber security and the internet of things: Vulnerabilities, threats, intruders and attacks. J. Cyber Secur. Mobil..

[89] Rohokale V., Prasad R. (2015). Cyber security for intelligent world with Internet of Things and machine to machine communication. J. Cyber Secur. Mobil..

[90] Saadeh M., Sleit A., Qatawneh M., Almobaideen W. Authentication techniques for the internet of things: A survey. Proceedings of the 2016 Cybersecurity and Cyberforensics Conference (CCC).

[91] Sivanathan A., Loi F., Gharakheili H.H., Sivaraman V. Experimental evaluation of cybersecurity threats to the smart-home. Proceedings of the 2017 IEEE International Conference on Advanced Networks and Telecommunications Systems (ANTS).

[92] Neshenko N. (2018). A Network Telescope Approach for Inferring and Characterizing IoT Exploitations. Ph.D. Thesis.

[93] Ainane N., Ouzzif M., Bouragba K. Data security of smart cities. Proceedings of the 3rd International Conference on Smart City Applications.

[94] Vrabie C. IoT and its role in developing smart cities. Proceedings of the Information Systems: Research, Development, Applications, Education: 11th SIGSAND/PLAIS EuroSymposium 2018.

[95] Viswanadham Y.V., Jayavel K. (2022). Blockchain Implementation in IoT Privacy and Cyber Security Feasibility Study and Analysis. High Performance Computing and Networking: Select Proceedings of CHSN 2021.

[96] Lewis M. (2018). Using Graph Databases to Assess the Security of Thingernets Based on the Thingabilities and Thingertivity of Things.

[97] Wu F.J., Solmaz G., Kovacs E. Toward the Future World of Internet-of-Things. Proceedings of the 2018 Global Internet of Things Summit (GIoTS).

[98] James F. IoT cybersecurity based smart home intrusion prevention system. Proceedings of the 2019 3rd Cyber Security in Networking Conference (CSNet).

[99] Shokeen R., Shanmugam B., Kannoorpatti K., Azam S., Jonkman M., Alazab M. Vulnerabilities analysis and security assessment framework for the internet of things. Proceedings of the 2019 Cybersecurity and Cyberforensics Conference (CCC).

[100] Roukounaki A., Efremidis S., Soldatos J., Neises J., Walloschke T., Kefalakis N. Scalable and configurable end-to-end collection and analysis of IoT security data: Towards end-to-end security in IoT systems. Proceedings of the 2019 Global IoT Summit (GIoTS).

[101] Van Kleek M., Seymour W., Binns R., Zhao J., Karandikar D., Shadbolt N. (2019). IoT Refine: Making Smart Home Devices Accountable for Their Data Harvesting Practices.

[102] Thorburn R., Margheri A., Paci F. (2019). Towards an Integrated Privacy Protection Framework for IoT: Contextualising Regulatory Requirements with Industry Best Practices.

[103] Nwafor E., Olufowobi H. Towards an Interactive Visualization Framework for IoT Device Data Flow. Proceedings of the 2019 IEEE International Conference on Big Data (Big Data).

[104] Ullah F., Naeem H., Jabbar S., Khalid S., Latif M.A., Al-Turjman F., Mostarda L. (2019). Cyber security threats detection in internet of things using deep learning approach. IEEE Access.

[105] Sharma R., Mahapatra R.P., Sharma N. (2020). The internet of things and its applications in cyber security. A Handbook of Internet of Things in Biomedical and Cyber Physical System.

[106] Karie N.M., Sahri N.M., Haskell-Dowland P. IoT threat detection advances, challenges and future directions. Proceedings of the 2020 workshop on emerging technologies for security in IoT (ETSecIoT).

[107] Andrade R.O., Yoo S.G., Tello-Oquendo L., Ortiz-Garcés I. (2020). A Comprehensive Study of the IoT Cybersecurity in Smart Cities. IEEE Access.

[108] Singh D., Pati B., Panigrahi C.R., Swagatika S. (2020). Security issues in IoT and their countermeasures in smart city applications. Advanced Computing and Intelligent Engineering: Proceedings of ICACIE 2018.

[109] Cvitić I., Perakovic D., Gupta B.B., Choo K.K.R. (2021). Boosting-based DDoS detection in internet of things systems. IEEE Internet Things J..

[110] Jhanjhi N., Humayun M., Almuayqil S.N. (2021). Cyber Security and Privacy Issues in Industrial Internet of Things. Comput. Syst. Sci. Eng..

[111] Strecker S., Van Haaften W., Dave R. An analysis of IoT cyber security driven by machine learning. Proceedings of the International Conference on Communication and Computational Technologies: ICCCT 2021.

[112] Ahmed K.D., Askar S. (2021). Deep learning models for cyber security in IoT networks: A review. Int. J. Sci. Bus..

[113] Houichi M., Jaidi F., Bouhoula A. A systematic approach for IoT cyber-attacks detection in smart cities using machine learning techniques. Proceedings of the Advanced Information Networking and Applications: Proceedings of the 35th International Conference on Advanced Information Networking and Applications (AINA-2021), Toronto, ON, Canada, 12–14 May 2021.

[114] Bhargava A., Salunkhe G., Bhargava S., Goswami P. (2021). A Comprehensive Study of IoT Security Risks in Building a Secure Smart City. Digit. Cities Roadmap IOT-Based Archit. Sustain..

[115] Al Solami E. (2021). Replication-aware secure resource administration scheme for Internet of Things-smart city applications. Trans. Emerg. Telecommun. Technol..

[116] Hulicki Z., Hulicki M. Cyber Security Aspects of Digital Services Using IoT Appliances. Proceedings of the Recent Challenges in Intelligent Information and Database Systems: 13th Asian Conference, ACIIDS 2021.

[117] Ali R.F., Muneer A., Dominic P., Taib S.M., Ghaleb E.A. Internet of things (IoT) security challenges and solutions: A systematic literature review. Proceedings of the Advances in Cyber Security: Third International Conference, ACeS 2021.

[118] Debnath D., Chettri S.K. Internet of Things: Current Research, Challenges, Trends and Applications. Proceedings of the Applications of Artificial Intelligence in Engineering: Proceedings of First Global Conference on Artificial Intelligence and Applications (GCAIA 2020), Jaipur, India, 8–10 September 2020.

[119] Toutsop O., Das S., Kornegay K. Exploring The Security Issues in Home-Based IoT Devices Through Denial of Service Attacks. Proceedings of the 2021 IEEE SmartWorld, Ubiquitous Intelligence & Computing, Advanced & Trusted Computing, Scalable Computing & Communications, Internet of People and Smart City Innovation (SmartWorld/SCALCOM/UIC/ATC/IOP/SCI).

[120] Balaji S., Jaishanker A., Gokhale S., Sinhal S., Rajeshkumar M. A Review on Cybersecurity of Internet of Things. Proceedings of the Microelectronic Devices, Circuits and Systems: Second International Conference, ICMDCS 2021.

[121] Khan M.A. (2022). A formal method for privacy-preservation in cognitive smart cities. Expert Syst..

[122] Nakkeeran M., Mathi S. (2021). A Generalized Comprehensive Security Architecture Framework for IoT Applications Against Cyber-Attacks. Artificial Intelligence and Technologies: Select Proceedings of ICRTAC-AIT 2020.

[123] Kowta A.S.L., Harida P., Venkatraman S.V., Das S., Priya V. Cyber Security and the Internet of Things: Vulnerabilities, Threats, Intruders, and Attacks. Proceedings of the International Conference on Computational Intelligence and Data Engineering: ICCIDE 2021.

[124] Maidamwar P.R., Bartere M.M., Lokulwar P.P. Implementation of network intrusion detection system using artificial intelligence: Survey. Proceedings of the 2nd International Conference on Recent Trends in Machine Learning, IoT, Smart Cities and
Applications: ICMISC 2021.

[125] Raimundo R.J., Rosário A.T. (2022). Cybersecurity in the Internet of Things in Industrial Management. Appl. Sci..

[126] Fan J., Yang W., Lam K.Y. (2022). Cybersecurity Challenges Of IoT-enabled Smart Cities: A Survey. arXiv.

[127] Ryu J.H., Moon S.Y., Park J.H. (2017). The study on data of smart home system as digital evidence. Advances in Computer Science and Ubiquitous Computing.

[128] Awasthi A., Read H.O., Xynos K., Sutherland I. (2018). Welcome pwn: Almond smart home hub forensics. Digit. Investig..

[129] Brotsis S., Kolokotronis N., Limniotis K., Shiaeles S., Kavallieros D., Bellini E., Pavué C. Blockchain solutions for forensic evidence preservation in IoT environments. Proceedings of the 2019 IEEE Conference on Network Softwarization (NetSoft).

[130] Iqbal A., Olegård J., Ghimire R., Jamshir S., Shalaginov A. Smart Home Forensics: An Exploratory Study on Smart Plug Forensic Analysis. Proceedings of the 2020 IEEE International Conference on Big Data (Big Data).

[131] Kim S., Park M., Lee S., Kim J. (2020). Smart home forensics—Data analysis of IoT devices. Electronics.

[132] Feng X., Dawam E.S., Amin S. Digital forensics model of smart city automated vehicles challenges. Proceedings of the Bigdata-2017.

[133] Hossain M.M., Hasan R., Zawoad S. Trust-IoV: A Trustworthy Forensic Investigation Framework for the Internet of Vehicles (IoV). Proceedings of the ICIOT.

[134] Zhang M., Zhou J., Cong P., Zhang G., Zhuo C., Hu S. (2022). LIAS: A Lightweight Incentive Authentication Scheme for Forensic Services in IoV. IEEE Trans. Autom. Sci. Eng..

[135] Tyagi R., Sharma S., Mohan S. Blockchain Enabled Intelligent Digital Forensics System for Autonomous Connected Vehicles. Proceedings of the 2022 International Conference on Communication, Computing and Internet of Things (IC3IoT).

[136] Zia T., Liu P., Han W. Application-specific digital forensics investigative model in internet of things (iot). Proceedings of the 12th International Conference on Availability, Reliability and Security.

[137] Rizal R., Riadi I., Prayudi Y. (2018). Network forensics for detecting flooding attack on internet of things (IoT) device. Int. J. Cyber-Secur. Digit. Forensics.

[138] Hou J., Li Y., Yu J., Shi W. (2019). A survey on digital forensics in Internet of Things. IEEE Internet Things J..

[139] Jayakrishnan A., Vasanthi V. Forensic Analysis on IoT Devices. Proceedings of the International Conference on Intelligent Data Communication Technologies and Internet of Things.

[140] Qatawneh M., Almobaideen W., Khanafseh M., Al Qatawneh I., Al-Ain P. (2019). Dfim: A New digital forensics investigation model for internet of things. J. Theor. Appl. Inf. Technol..

[141] Yaqoob I., Hashem I.A.T., Ahmed A., Kazmi S.A., Hong C.S. (2019). Internet of things forensics: Recent advances, taxonomy, requirements, and open challenges. Future Gener. Comput. Syst..

[142] Stoyanova M., Nikoloudakis Y., Panagiotakis S., Pallis E., Markakis E.K. (2020). A survey on the internet of things (IoT) forensics: Challenges, approaches, and open issues. IEEE Commun. Surv. Tutorials.

[143] Patil S.S., JCER B., Dinesha H., SGBIT B. (2020). Secure Cyber Forensic Frameworks for Internet of Things. Int. J. Eng. Appl. Sci. Technol..

[144] Jayakrishnan A., Vasanthi V. Internet of things forensics honeynetcloud investigation model. Proceedings of the 2020 International Conference on Electronics and Sustainable Communication Systems (ICESC).

[145] Atlam H.F., Hemdan E.E.D., Alenezi A., Alassafi M.O., Wills G.B. (2020). Internet of things forensics: A review. Internet Things.

[146] Patel R., Malek Z. (2020). Brief overview of existing challenges in IoT. Int. J. Emerg. Trends Technol. Computer Sci. (IJETTCS).

[147] Yang W., Johnstone M.N., Sikos L.F., Wang S. Security and forensics in the internet of things: Research advances and challenges. Proceedings of the 2020 Workshop on Emerging Technologies for Security in IoT (ETSecIoT).

[148] Bandil A., Al-Masri E. VTA-IH: A Fog-based Digital Forensics Framework. Proceedings of the 2020 6th International Conference on Science in Information Technology (ICSITech).

[149] Janarthanan T., Bagheri M., Zargari S. (2021). IoT forensics: An overview of the current issues and challenges. Digital Forensic Investigation of Internet of Things (IoT) Devices.

[150] Surange G., Khatri P. IoT forensics: A review on current trends, approaches and foreseen challenges. Proceedings of the 2021 8th International Conference on Computing for Sustainable Global Development (INDIACom).

[151] Kim S., Jo W., Lee J., Shon T. (2022). AI-enabled device digital forensics for smart cities. J. Supercomput..

[152] Ganesh N., Venkatesh N., Prasad D. (2022). A Systematic Literature Review on Forensics in Cloud, IoT, AI & Blockchain. Illumination of Artificial Intelligence in Cybersecurity and Forensics.

[153] Sharma J., Singh M. (2015). Web Services Oriented Architecture for DPI based Network Forensics Grid. Int. J. Energy, Inf. Commun..

[154] Mishra A., Singh C., Dwivedi A., Singh D., Biswal A.K. Network Forensics: An approach towards detecting Cyber Crime. Proceedings of the 2021 International Conference in Advances in Power, Signal, and Information Technology (APSIT).

